# The Objects and Limits of Operations for Cancer

**Published:** 1897-03

**Authors:** 


**The Objects and Limits of Operations for Cancer.** By W. Watson Cheyne, M.B., F.R.S. Pp. vi., 140. London: Baillière, Tindall, and Cox. 1896.

This book, which consists of the Lettsomian Lectures for 1896, reprinted from the *Transactions of the Medical Society of London*, is one which should be carefully studied by every surgeon—and, indeed, by every medical practitioner. The regions affected with cancerous growths, referred to by Mr. Watson Cheyne, are the breast, the throat and tongue, and the intestine.

In dealing with the surgery of breast carcinoma, he first describes the result of recent investigations into the anatomy of the breast and its lymphatic system, and points out their bearing on its operative treatment. He has strong condemnation for any partial operation such as removal of the breast without also clearing out the axilla, even in cases in which no axillary glands can be felt; and he advises that the subcutaneous fatty tissue around the breast should be removed as far inwards as the sternum and as far upwards as the clavicle, by freely undermining the skin. By thorough removal of the disease, Mr. Watson Cheyne contends that now it is not a question simply of prolongation of life, but in an ever increasing number of cases, of cure of the disease, and his own statistics of operation for cancer of the breast bear out his contention. He does not hesitate to remove a portion of the axillary vein when it is adherent to a mass of axillary glands, and has even removed on one occasion a portion of both artery and vein without any loss of vitality in the arm; but he does not agree with the recommendation of Mr. Arbuthnot Lane to divide the clavicle and clear out the supra-clavicular glands when they are involved, as he believes that the disease will then probably have reached the mediastinal set of glands and be ineradicable.

With regard to cancer of the tongue, Mr. Watson Cheyne lays stress on the advantage of removal of the tongue close to the hyoid bone, by Kocher's operation, in cases of deep infiltration of the organ, and of the removal of the glandular area in the submaxillary region, in the same way that we clear out the axilla in operating for cancer of the breast.

We consider that perhaps the most important part of the book is that which relates to operations for malignant growths in the throat. Of course such operations are always serious and the mortality attending them high; but the operations undertaken by Mr. Watson Cheyne for this terrible disease have been attended by a surprising amount of success considering their very formidable character. He not only gives us his own experience of these operations—undertaken both for epithelioma involving the throat and tongue and for sarcoma of the tonsils,—but also a most elaborate and extremely valuable table of the operations performed in such cases by other surgeons. When operating by external incision, his experience has been that ligation of the external carotid artery is very liable to be followed by secondary hemorrhage, as the wound is of course septic owing to its free communication with the mouth; and hence he prefers to divide the jaw and thus get free access to the tonsillar region for the purpose of readily dealing with all the bleeding vessels. Enlarged glands in the neck, unless they involve the vagus or carotid artery, he does not consider a contra-indication to operation, as they can be freely removed, together with the internal jugular vein, if they are adherent to it.

Mr. Watson Cheyne does not go so fully into the subject of intestinal cancer as he does into that of malignant disease of the breast or throat, but he contrasts the advantages to be derived from the palliative operation of colotomy with those obtained by excision of rectal cancers. He agrees with what he considers the views of the English school of surgery as opposed to the German, that it would be better to exclude from the radical operation cases of rapid growth where the disease forms a large ulcerated mass, and those in which it has extended through the rectal wall and invaded surrounding parts, or is situated high up, though limited to the bowel.

Our outline of the contents of this book will be enough to indicate its great value to every operating surgeon, especially as it is written in Mr. Watson Cheyne's lucid and forcible manner, and as the outcome of the author's experience in the operative treatment of malignant disease.

We are at a loss to understand the absence of an index and the presence of forty-two pages of advertisements.

